# Supplementary material for: Neutrophil metalloproteinase driven spleen damage hampers infection control of trypanosomiasis
Source: Nat Commun. 2023 Sep 5;14:5418. doi: 10.1038/s41467-023-41089-w (PMC10480172; doi:10.1038/s41467-023-41089-w)
Supplement: Supplementary file 1 — Supplementary Information [file 41467_2023_41089_MOESM1_ESM.pdf]

Supplementary Table s1. Antibodies for flow cytometry

Mouse antibodies	Clone	Company	Catalog number	Dilution
Alexa Fluor ® 488 anti-mouse Ly-6G	1A8	BioLegend	127626	1/600
PE anti-mouse Ly-6G	1A8	BioLegend	127607	1/600
PE/Cyanine7 anti-mouse Ly-6C	HK1.4	BioLegend	128017	1/600
FITC anti-mouse/human CD11b	M1/70	BioLegend	101206	1/600
APC anti-mouse/human CD11b	M1/70	BioLegend	101212	1/600
PE anti-mouse CD182 (CXCR2)	SA044G4	BioLegend	149303	1/100
PE anti-mouse CD184 (CXCR4)	L276F12	BioLegend	146505	1/100
Alexa Fluor® 647 rat anti-mouse CD177	Y127	BD Biosciences	566599	1/600
FITC anti-mouse CD4	GK1.5	BioLegend	100406	1/600
PE anti-mouse CD8a	53-6.7	BioLegend	100708	1/600
APC anti-mouse NK-1.1	PK136	BioLegend	108710	1/600
FITC anti-mouse/human CD45R/B220	RA3-6B2	BioLegend	103206	1/600
PE anti-mouse/human CD45R/B220	RA3-6B2	BioLegend	103208	1/600
PE anti-mouse CD1d (CD1.1, Ly-38)	1B1	BioLegend	123510	1/600
PE anti-mouse IgM	RMM-1	BioLegend	406507	1/600
PE/Cyanine7 anti-mouse CD138 (Syndecan-1)	281-2	BioLegend	142514	1/600
PE/Cyanine7 anti-mouse CD93 (AA4.1, early B lineage)	AA4.1	BioLegend	136506	1/600
APC anti-mouse CD93 (AA4.1, early B lineage)	AA4.1	BioLegend	136510	1/600
APC anti-mouse CD23	B3B4	BioLegend	101619	1/600
APC anti-mouse CD19	1D3/CD19	BioLegend	152409	1/600
FITC anti-mBCMA	161616	R&D System	TAB593F	1/100

Supplementary Table s2. Antibodies for immuno-fluorescence and immuno-histochemistry

Antibodies	Clone	Company	Catalog number	Note	Dilution
Alexa Fluor ® 488 anti-mouse Ly-6G	1A8	BioLegend	127626	Primary antibody	1/20
Anti-Myeloperoxidase antibody	2D4	abcam	ab90810	Primary antibody (biotin conjugated)	1/200
Neutrophil Elastase Polyclonal Antibody, Phycoerythrin Conjugated		Bioss	BS-6982R-PE	Primary antibody	1/200
Recombinant Anti-Histone H2B antibody	EP957Y	abcam	ab52599	Primary antibody	1/40
Rabbit anti Mouse Collagen I		Bio-Rad	2150-1410	Primary antibody	1/50
Elastin Polyclonal Antibody		Bioss	BS-1756R	Primary antibody	1/200
Cyanine3 Streptavidin		BioLegend	405215	Secondary antibody for MPO	1/1000
Goat Anti-Rabbit IgG H&L (Alexa Fluor® 488)		abcam	ab150077	Secondary antibody for Histone H2B and Collagen	1/200
DAPI		Sigma-Aldrich		Nucleus staining (Counter staining)	1/1000

Supplementary Table s3: Quality Control (QC) Metrics.

Detailed summary of the quality control metrics for the scRNA-seq data obtained from spleen and bone marrow, at various stages of the data processing pipeline, organized into four key steps: (1) initial output of cells after processing with CellRanger Count, (2) cells remaining after the removal of ambient RNA using SoupX, (3) cells retained after eliminating technical noise, and (4) final selection of cells after applying filtering criteria in Seurat. The QC metrics are presented for each sample group, enabling a thorough evaluation of data quality and uniformity throughout the entire study.

			**Naïve**	**Naïve**	***T. brucei* 14dpi**	***T. brucei* 14dpi**
	**Tissue**		Spleen	Bone marrow	Spleen	Bone marrow
**Cell Ranger Count Output**	**Cells**	Estimated Number of Cells	9,991	8,579	10,619	8,020
		Fraction Reads in Cells	80.70%	95.0%	93.10%	95.3%
		Mean Reads per Cell	55,468	19,605	49,601	20,141
		Median Genes per Cell	1,734	1,969	1,655	1,716
		Total Genes Detected	19,477	22,344	20,507	21,992
		Median UMI Counts per Cell	4,362	5,708	5,469	5,444
	**Sequencing**	Number of Reads	554,177,373	168,189,439	526,709,533	161,529,396
		Number of Short Reads Skipped	0	0	0	0
		Valid Barcodes	96.00%	98.2%	97.00%	98.2%
		Valid UMIs	99.90%	99.9%	99.90%	99.9%
		Sequencing Saturation	77.90%	43.1%	64.20%	39.1%
		Q30 Bases in Barcode	95.80%	95.7%	95.60%	95.7%
		Q30 Bases in RNA Read	92.30%	93.5%	93.30%	92.9%
		Q30 Bases in UMI	92.80%	92.5%	92.70%	92.5%
	**Mapping**	Reads Mapped to genome	94.60%	97.0%	96.20%	96.7%
		Reads Mapped Confidently to Genome	90.70%	94.4%	91.30%	94.0%
		Reads Mapped Confidently to Intergenic Regions	5.50%	3.1%	3.20%	3.0%
		Reads Mapped Confidently to Introgenic Regions	27.30%	19.4%	13.90%	16.5%
		Reads Mapped Confidently to Exogenic Regions	57.90%	71.9%	74.20%	74.5%
		Reads Mapped Confidently to Transcriptome	55.40%	85.1%	71.70%	85.4%
		Reads Mapped Antisense to Gene	1.40%	5.8%	1.30%	5.3%
	**Sample**	Chemistry	Single Cell 3' v3	Single Cell 3' v3	Single Cell 3' v3	Single Cell 3' v3
		Transcriptome	mm10-2020-A	mm10-2020-A	mm10-2020-A	mm10-2020-A
		Pipeline Version	cellranger-5.0.0	cellranger-7.0.1	cellranger-5.0.0	cellranger-7.0.1
**Ambient RNA Removal**		Number of cells	9,881	8,579	9,685	8,020
		Means Reads per Cells	4,896	8,681	12,092	9,663
		Median Reads per Cells	4,228	5,604	6,180	5,365
		Means Genes per Cell	1,652	2,394	2,126	2,384
		Median Genes per Cell	1,753	1,966	1,857	1,712
**Removal of technical noise Gm42418 and AY036118**		Number of cells	9,881	8,579	9,685	8,020
		Means Reads per Cells	4,783	8,651	12,031	9,639
		Median Reads per Cells	4,123	5,579	6,105	5,346
		Means Genes per Cell	1,650	2,392	2,123	2,382
		Median Genes per Cell	1,751	1,964	1,854	1,711
**Filtering in Seurat**		Threshold	200 < nFeature < 4000 mito genes > 10%	100 < nFeature < 6000 mito genes > 10%	200 < nFeatur e< 5000 mito genes > 10%	100 < nFeature < 6000 mito genes > 10%
		Number of cells	6,375	7,951	7,969	7,352
		Means Reads per Cells	5,882	8,098	8,837	8,500
		Median Reads per Cells	5,699	5,697	6,026	5,307
		Means Genes per Cell	1,995	2,378	1,873	2,275
		Median Genes per Cell	2,134	1,986	1,838	1,695

Supplementary Table s4. The list of DEGs between 14 dpi mice and naïve controls. Differential expressed genes DEGs) in the N4 subcluster between the 14 dpi and naïve datasets were identified as described in the Methods section. The Wilcoxon rank sum test was used for statistical analysis with *p*-values adjusted using the Bonferroni correction, considering the total number of genes in the dataset.

	avg_log2FC	p_val_adj
**H2-Q7**	2.95	3.73E-19
**H2-D1**	1.23	1.80E-13
**S100a9**	1.64	4.32E-13
**Ifitm3**	1.93	2.61E-12
**Gbp2**	3.18	4.44E-12
**H2-K1**	1.17	1.15E-11
**Txn1**	1.25	1.24E-11
**Ly6c2**	3.46	2.87E-10
**S100a8**	1.43	6.92E-10
**Rps29**	-1.77	1.18E-09
**H2-Q6**	1.85	1.59E-09
**Rps21**	-1.62	2.32E-09
**Lyz2**	1.99	1.46E-08
**Retnlg**	2.33	8.17E-08
**Gbp7**	1.99	1.49E-07
**Mgst1**	2.10	1.68E-07
**Cd74**	-1.28	1.72E-07
**Prdx5**	1.26	2.56E-07
**H2-T22**	1.47	5.07E-07
**S100a6**	1.22	6.34E-07
**Ubb**	1.11	9.37E-07
**Stat1**	1.34	1.03E-06
**Junb**	-0.95	4.29E-06
**H2-T23**	1.27	5.08E-06
**Ighg2c**	1.44	6.86E-06
**Igtp**	1.85	1.60E-05
**Psmb9**	1.10	2.25E-05
**Iglc2**	-1.59	2.31E-05
**Cd52**	0.80	2.32E-05
**Rplp2**	-1.47	2.82E-05
**Ifit3**	2.30	3.14E-05
**Ly6g**	2.34	3.29E-05
**Ighg1**	1.32	3.33E-05
**Upp1**	2.05	7.52E-05
**Isg15**	1.87	1.07E-04
**Rtp4**	1.08	1.49E-04
**Zbp1**	1.56	2.20E-04
**Gapdh**	0.99	3.13E-04

	avg_log2FC	p_val_adj
**Ifi204**	1.79	4.24E-04
**Marcks**	-1.01	4.72E-04
**Rpl37a**	-1.19	5.13E-04
**Dusp1**	-1.02	7.79E-04
**Dazap2**	-1.27	7.87E-04
**Rps26**	-1.85	9.69E-04
**H2-Q4**	0.94	1.25E-03
**Cd79a**	-1.19	1.32E-03
**Ifi27l2a**	1.19	1.37E-03
**Rpl38**	-1.50	1.38E-03
**Ngp**	2.52	1.49E-03
**Grk2**	-1.33	1.54E-03
**Itgb7**	1.40	1.77E-03
**Slfn4**	0.70	1.92E-03
**Irf7**	1.33	2.08E-03
**Il1rap**	-1.38	3.76E-03
**Itm2b**	0.70	3.84E-03
**Irgm1**	1.56	3.97E-03
**Prr13**	0.80	4.05E-03
**Camp**	2.27	4.56E-03
**Rps28**	-1.68	5.49E-03
**Lgals3**	1.34	5.81E-03
**Mmp8**	1.70	5.98E-03
**Jund**	-0.86	6.18E-03
**Glrx**	1.24	6.89E-03
**Pglyrp1**	0.97	7.71E-03
**Crip1**	-1.35	1.05E-02
**Sdc4**	-1.02	1.12E-02
**Rps27**	-0.88	1.91E-02
**Dhx9**	-0.83	2.22E-02
**Psmb8**	0.99	2.25E-02
**Ifi47**	1.55	2.54E-02
**1600014C10Rik**	1.18	3.10E-02
**Samhd1**	0.77	3.61E-02
**Gbp4**	1.79	4.34E-02
**Rpl35a**	-1.13	4.83E-02
**Nr4a1**	-1.45	4.84E-02
**Timp2**	0.98	4.90E-02

Figure S1

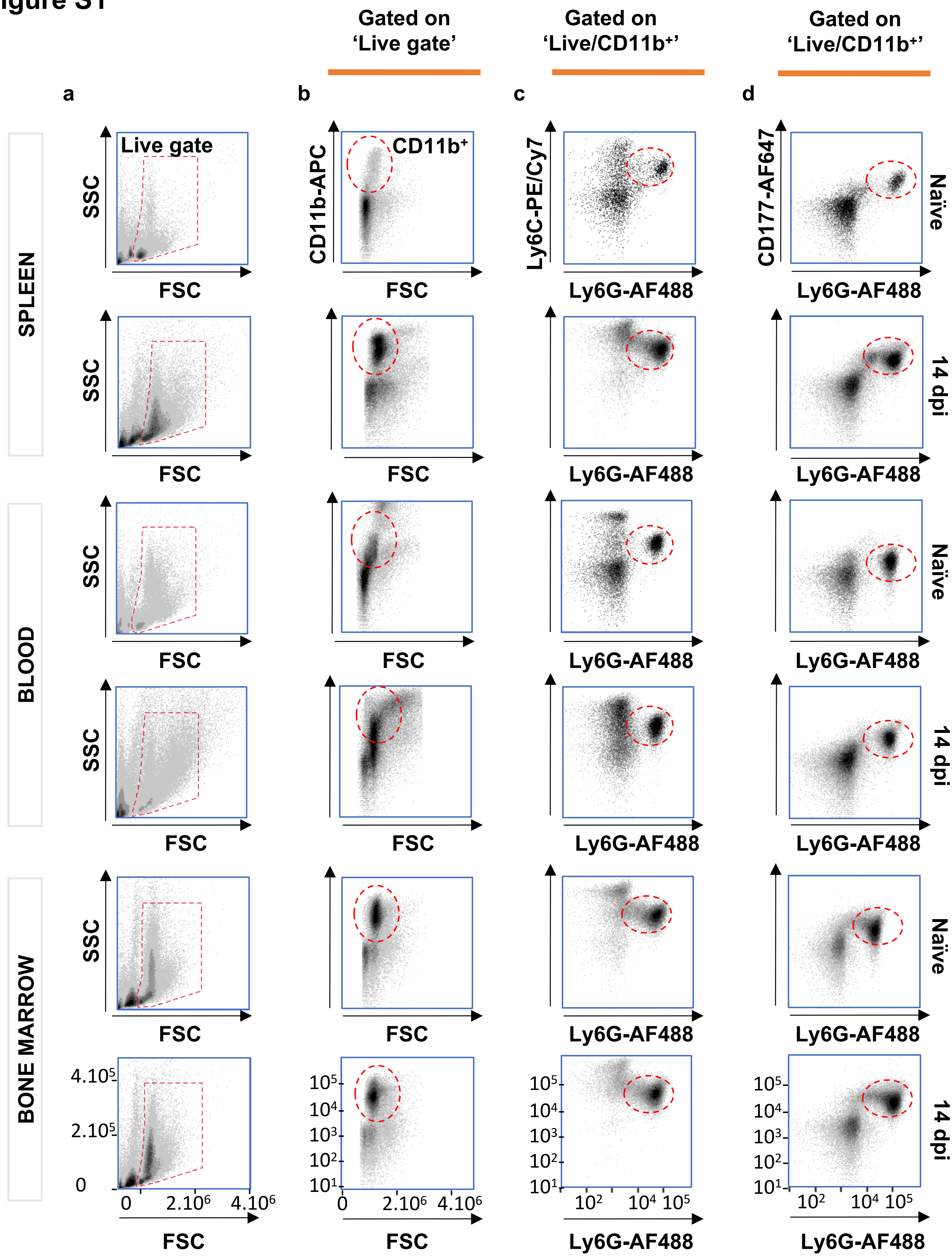
Gated on 'Live gate'
Gated on 'Live/CD11b⁺'
Gated on 'Live/CD11b⁺'
a
b
c
d
SPLEEN
BLOOD
BONE MARROW
Live gate
CD11b⁺
SSC
FSC
CD11b-APC
Ly6C-PE/Cy7
Ly6G-AF488
CD177-AF647
Naïve
14 dpi
4.10⁵
2.10⁵
0
2.10⁶
4.10⁶
10⁵
10⁴
10³
10²
10¹
10²
10⁴ 10⁵

Supplementary Fig. s1. Representative flow cytometry gating strategy for neutrophils. From top to bottom samples represent spleen, blood and bone marrow samples from naive and 14dpi mice. From left to right sample columns represent **a** FSC/SSC to eliminate debris and red blood cells from the 'Live gate'. **b** FSC/CD11b to define the CD11b$^+$ cells population, gated in the 'Live gate'. **c** Ly6G/Ly6C plots showing the specific area for neutrophils, gated on the "Live gated CD11b$^+$ cells", with neutrophils defined as the Ly6G$^+$Ly6C$^+$ population. **d** Ly6G/CD177 plots for neutrophils, gated on the "Live CD11b$^+$ cells". Scaling of axes for all graphs in each column is indicated in the lower panel.

Figure S2

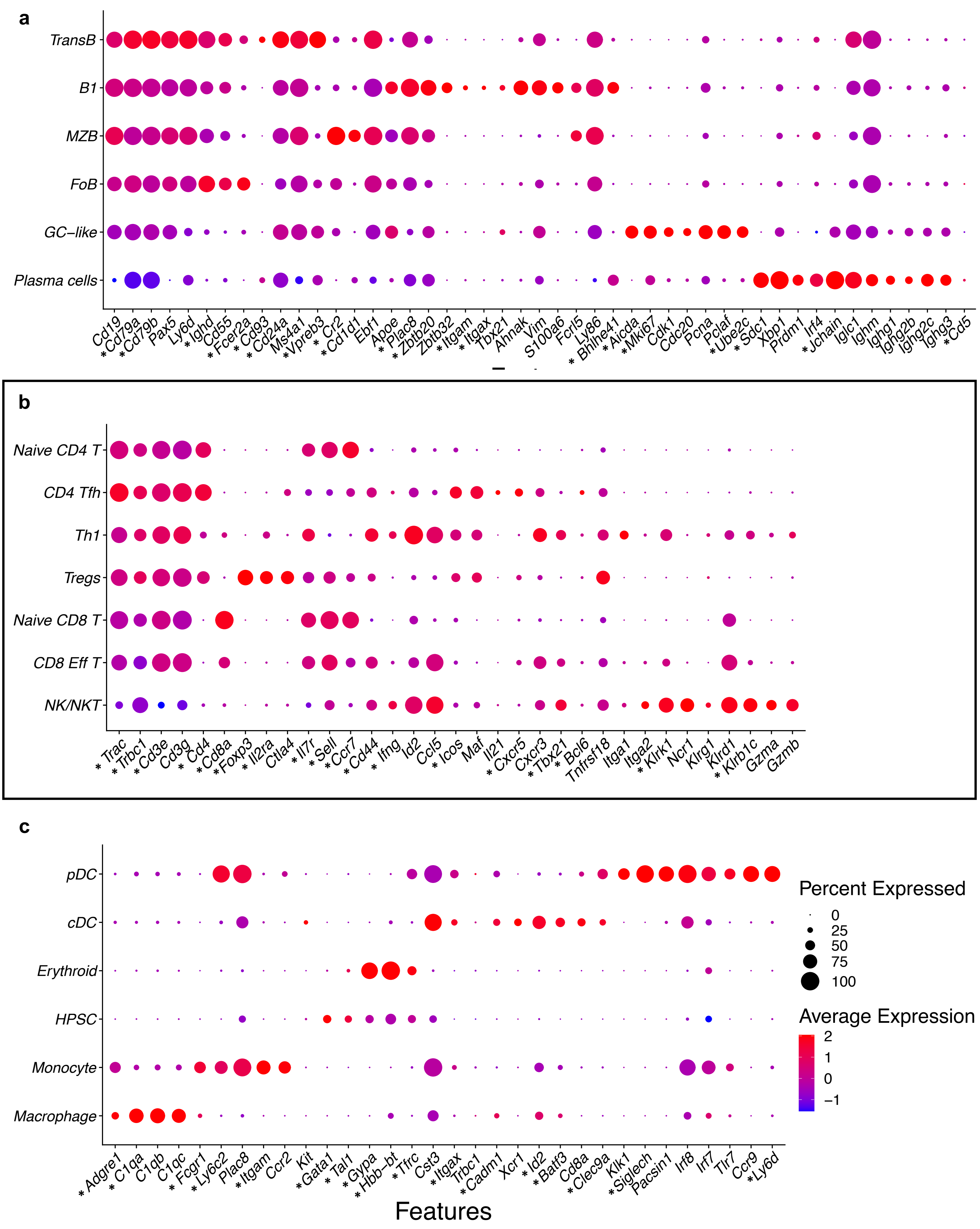
a
TransB
B1
MZB
FoB
GC−like
Plasma cells
Cd19
*Cd79a
*Cd79b
Pax5
Ly6d
*Ighd
Cd55
*Fcer2a
*Cd93
*Cd24a
Ms4a1
*Vpreb3
*Cr2
*Cd1d1
Ebf1
Apoe
*Plac8
*Zbtb20
Zbtb32
*Itgam
*Itgax
Tbx21
Ahnak
Vim
S100a6
Fcrl5
Ly86
*Bhlhe41
*Aicda
*Mki67
Cdk1
Cdc20
Pcna
Pclaf
*Ube2c
*Sdc1
Xbp1
Prdm1
Irf4
*Jchain
Iglc1
Ighm
Ighg1
Ighg2b
Ighg2c
Ighg3
*Cd5
b
Naive CD4 T
CD4 Tfh
Th1
Tregs
Naive CD8 T
CD8 Eff T
NK/NKT
*Trac
*Trbc1
*Cd3e
Cd3g
*Cd4
*Cd8a
*Foxp3
*Il2ra
Ctla4
*Il7r
*Sell
*Ccr7
*Cd44
*Ifng
Id2
Ccl5
*Icos
Maf
Il21
*Cxcr5
Cxcr3
*Tbx21
*Bcl6
Tnfrsf18
Itga1
Itga2
*Klrk1
Ncr1
Klrg1
Klrd1
*Klrb1c
Gzma
Gzmb
c
pDC
cDC
Erythroid
HPSC
Monocyte
Macrophage
*Adgre1
*C1qa
C1qb
C1qc
*Fcgr1
*Ly6c2
Plac8
*Itgam
Ccr2
Kit
*Gata1
*Tal1
*Gypa
*Hbb−bt
*Tfrc
Cst3
*Itgax
Trbc1
*Cadm1
Xcr1
*Id2
*Batf3
Cd8a
*Clec9a
Klk1
*Siglech
Pacsin1
Irf8
Irf7
Tlr7
Ccr9
*Ly6d
Features
Percent Expressed
0
25
50
75
100
Average Expression
2
1
0
−1

Supplementary Figure s2. **Spleen cell annotation according to signature gene expression** (5,6, 35-39). **a** Dot plot displaying the average expression of signature genes for *Cd79a*$^+$/*b*$^+$ B cells including *Cd93*$^+$*Cd24a*$^+$*Vpreb3*$^+$ Transitional B cells (TransB), *Zbtb20*$^+$*Plac8*$^+$*Itgam*$^-$*Cd5*$^-$ B1 cells, *Cr2*$^+$*Cd1d*$^+$*Fcer2a*$^+$ Marginal Zone B cells (MZB), *Ighd*$^+$*Fcer2a*$^+$*Cr2*$^-$ Follicular B cells (FoB), *Aicda*$^+$*Mki67*$^+$ Germinal Center – like (GC-like) B cells and *Sdc1*$^+$*Xbp1*$^+$*Jchain*$^+$ Plasma cells (PC). Dot size shows the percentage of cells in each subcluster with more than one read of the corresponding gene. **b** Dot plot displaying the average expression of signature genes for *Trac*$^+$ *Cd3e*$^+$ T cells including Naïve *Cd4*$^+$*Il7*$^+$*Ccr7*$^+$*Cd44*$^-$ T cells, *Cd4*$^+$*Cxcr5*$^+$*Icos*$^+$*Bcl6*$^+$ Follicular (Tfh) T cells, *Cd4*$^+$ *Ifng*$^+$*Tbx21*$^+$ T helper 1 cells (Th1), *Foxp3*$^+$*IL2ra*$^+$ regulatory T cells (Tregs), *Cd8a*$^+$*Il7*$^+$*Ccr7*$^+$*Cd44*$^-$*Sell*$^+$ naïve T cells, *Cd8a*$^+$*Il7*$^+$*Ccr7*$^+$*Cd44*$^+$*Sell*$^+$ Effector (Eff T). A mixed population of Natural Killer T cells (NKT)/Natural Killer cells was identified using combination of *Trac*, *Trbc1*, *Klrb1c*, *Klrk1*, and *Gzmb*. **c** Other cell populations were annotated as follows: *Siglech*$^+$*Ly6c2*$^+$ *Irf7*$^+$*Tlr7*$^+$*Pacsin1*$^+$ plasmacytoid Dendritic cells (pDC), *Cd8a*$^+$*Tbrc1*$^-$ *Xcr1*$^+$*Id2*$^+$ conventional Dendritic cells (cDC), *Gypa*$^+$*Tfrc*$^+$*Hbb-bt*$^+$ Erythroid cells, *Gata1*$^+$*Tal1*$^+$ Hematopoietic Stem Cells (HPSC), *Fcgr1*$^+$*Ly6c2*$^+$*Plac8*$^+$*Itgam*$^+$ Monocytes, and *Adegre1*$^+$*C1qa*$^+$*Ly6c2*$^-$*Itgam*$^-$ Macrophages. Dot size shows the percentage of cells in each subcluster with more than one read of the corresponding gene. Dot size indicates the percentage of cells within each subcluster expressing more than one read of the corresponding gene. Key markers genes used are highlighted using an indicator (*).

Figure S3

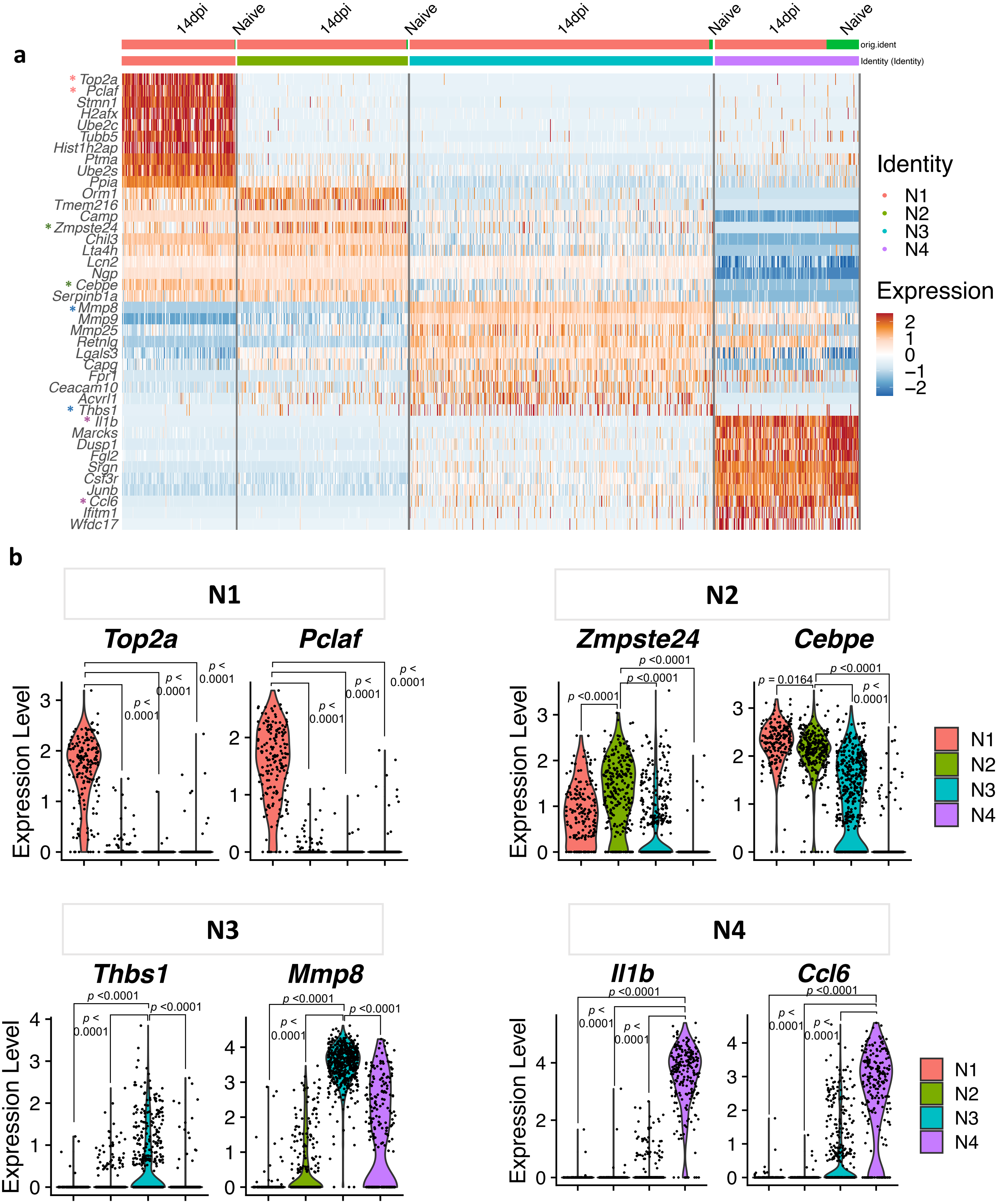

a
14dpi
Naive
orig.ident
Identity (Identity)
Top2a
Pclaf
Stmn1
H2afx
Ube2c
Tubb5
Hist1h2ap
Ptma
Ube2s
Ppia
Orm1
Tmem216
Camp
Zmpste24
Chil3
Lta4h
Lcn2
Ngp
Cebpe
Serpinb1a
Mmp8
Mmp9
Mmp25
Retnlg
Lgals3
Capg
Fpr1
Ceacam10
Acvrl1
Thbs1
Il1b
Marcks
Dusp1
Fgl2
Srgn
Csf3r
Junb
Ccl6
Ifitm1
Wfdc17
Identity
N1
N2
N3
N4
Expression
2
1
0
−1
−2
b
N1
Top2a
Pclaf
N2
Zmpste24
Cebpe
N3
Thbs1
Mmp8
N4
Il1b
Ccl6
Expression Level
p < 0.0001
p = 0.0164

Supplementary Fig. s3. Differential expression of top marker genes in spleen splenic neutrophils. **a** Heatmap showing expression of 10 highest DEGs, a Wilcoxon rank sun test was used. DEGs were defined as having a 0.58 < Log_2 Fold Change < 0.58, with adjusted *p*-values < 0.05. For every subpopulation, two key marker gene are highlighted with a color-coded indicator (*). **b** Violin plots representing differences in the key marker gene expression indicated above for N1/N2/N3/N4 neutrophil subpopulations. For comparisons of individual genes, *p*-values were used from the DEG analysis in Seurat. The Wilcoxon rank sum test was used for statistical analysis with *p*-values adjusted using the Bonferroni correction, with $p < 0.05$ considered statistically significant.

Figure S4

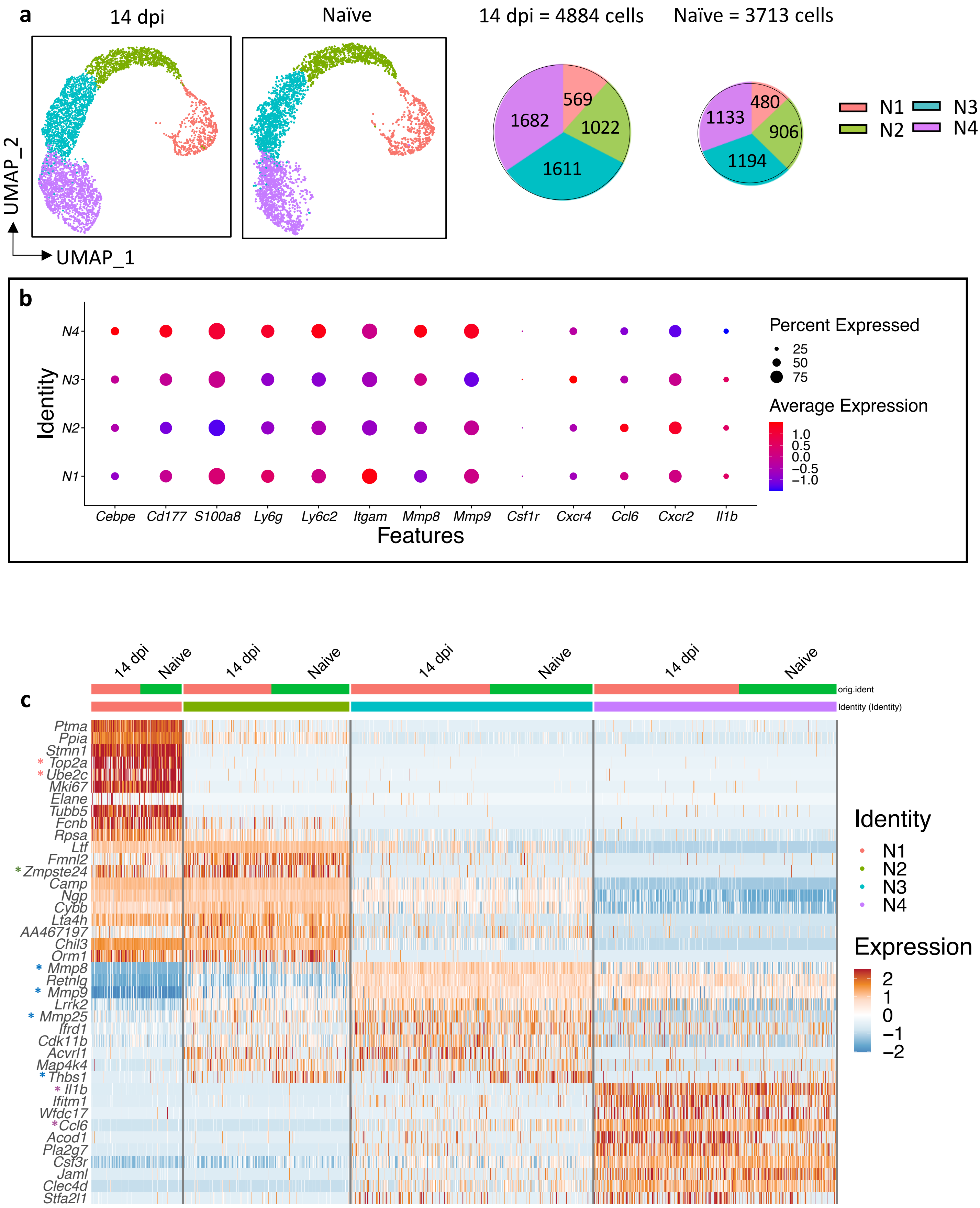

a
14 dpi
Naïve
UMAP_2
UMAP_1
14 dpi = 4884 cells
Naïve = 3713 cells
569
1022
1611
1682
480
906
1194
1133
N1
N2
N3
N4
b
N4
N3
N2
N1
Identity
Cebpe
Cd177
S100a8
Ly6g
Ly6c2
Itgam
Mmp8
Mmp9
Csf1r
Cxcr4
Ccl6
Cxcr2
Il1b
Features
Percent Expressed
25
50
75
Average Expression
1.0
0.5
0.0
−0.5
−1.0
c
14 dpi
Naive
orig.ident
Identity (Identity)
Ptma
Ppia
Stmn1
* Top2a
* Ube2c
Mki67
Elane
Tubb5
Fcnb
Rpsa
Ltf
Fmnl2
* Zmpste24
Camp
Ngp
Cybb
Lta4h
AA467197
Chil3
Orm1
* Mmp8
Retnlg
* Mmp9
Lrrk2
* Mmp25
Ifrd1
Cdk11b
Acvrl1
Map4k4
* Thbs1
* Il1b
Ifitm1
Wfdc17
* Ccl6
Acod1
Pla2g7
Csf3r
Jaml
Clec4d
Stfa2l1
Identity
N1
N2
N3
N4
Expression
2
1
0
−1
−2

Supplementary Fig. s4. Characterization of bone marrow (BM) neutrophil subpopulations in 14 dpi and naïve mice. a Uniform Manifold Approximation and Projection (UMAP) plot of neutrophils from 14 dpi mouse BM and naive mouse BM, colored by cluster identity. Neutrophils were clustered into four subpopulations (N1-N4). Pie chart demonstrating the proportions of four subpopulations in the total number of barcoded neutrophils from 14 dpi and naïve mice. **b** Dot plot illustrating the average expression of neutrophil marker genes across each neutrophil subcluster (N1-N4) using combined data of 14dpi and naïve mice. Neutrophil marker genes such as *Cebpe, Cd177*, *Ly6g*, *S100a8, Ly6c, Itgam, Cxcr4* and *Cxcr2* were used for annotation (40). Dot size represents the percentage of cells in each cluster with more than one read of the corresponding genes and dot color displays the average expression of each gene in each cluster. **c** Heatmap representing 10 highest DEGs among four subclusters (N1-N4) in 14 dpi and naïve mice. Genes having Log_2 Fold Change > 0.25 and adjusted *p-value* < 0.05 were defined as DEGs. Key markers genes used elsewhere in the spleen neutrophil analysis, are highlighted with a color-coded indicator (*).

Figure S5

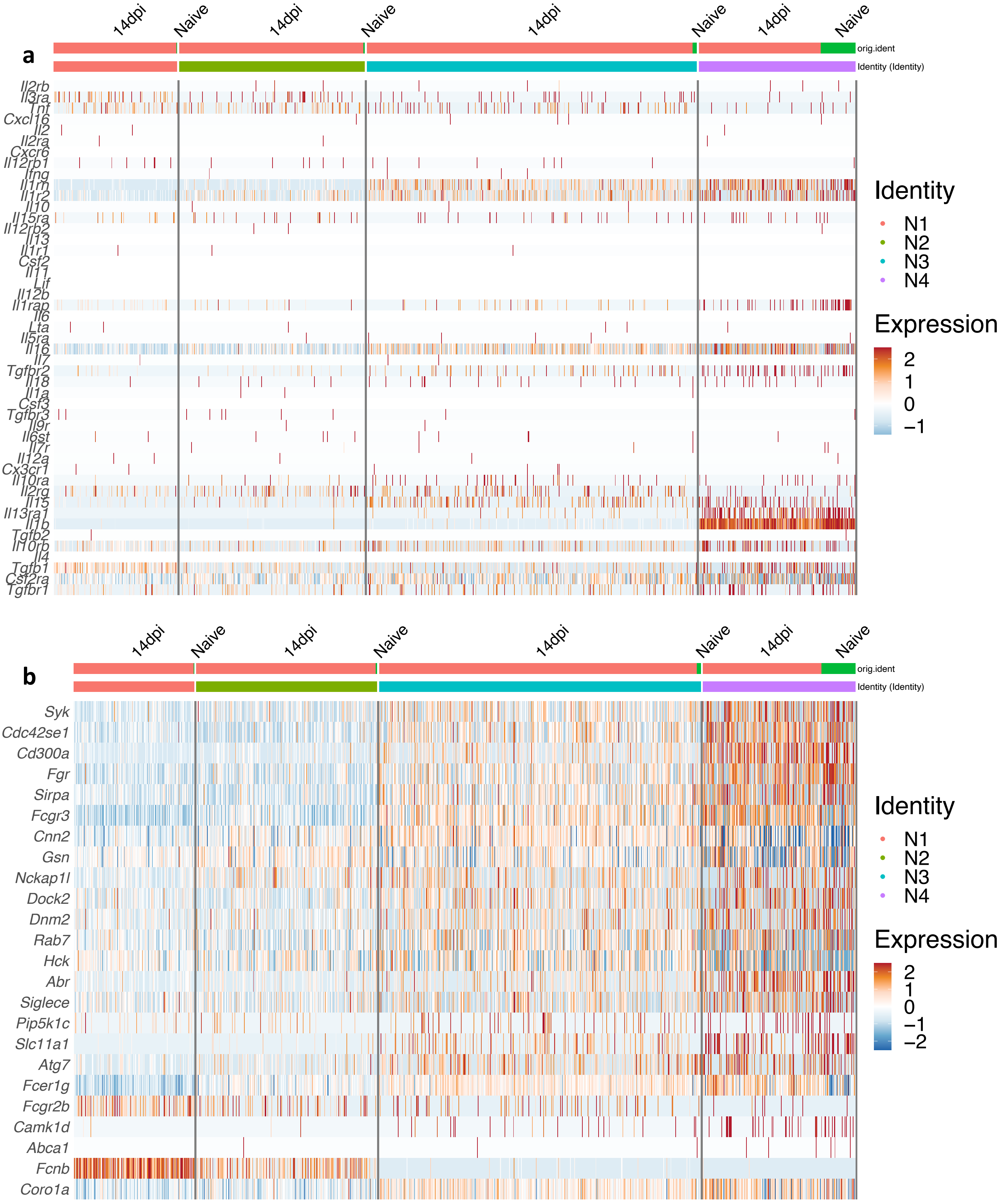
a
14dpi
Naive
14dpi
Naive
14dpi
Naive
14dpi
Naive
orig.ident
Identity (Identity)
Il2rb
Il3ra
Tnf
Cxcl16
Il2
Il2ra
Cxcr6
Il12rb1
Ifng
Il1rn
Il1r2
Il10
Il15ra
Il12rb2
Il13
Il1r1
Csf2
Il11
Lif
Il12b
Il1rap
Il6
Lta
Il5ra
Il16
Il7
Tgfbr2
Il18
Il1a
Csf3
Tgfbr3
Il9r
Il6st
Il7r
Il12a
Cx3cr1
Il10ra
Il2rg
Il15
Il13ra1
Il1b
Tgfb2
Il10rb
Il4
Tgfb1
Csf2ra
Tgfbr1
Identity
N1
N2
N3
N4
Expression
2
1
0
−1
b
14dpi
Naive
14dpi
Naive
14dpi
Naive
14dpi
Naive
orig.ident
Identity (Identity)
Syk
Cdc42se1
Cd300a
Fgr
Sirpa
Fcgr3
Cnn2
Gsn
Nckap1l
Dock2
Dnm2
Rab7
Hck
Abr
Siglece
Pip5k1c
Slc11a1
Atg7
Fcer1g
Fcgr2b
Camk1d
Abca1
Fcnb
Coro1a
Identity
N1
N2
N3
N4
Expression
2
1
0
−1
−2

Supplementary Fig. s5. a Row-scaled gene expression of marker genes (35-37) coding for cytokines and chemokines, per cluster in 14 dpi mice and naïve mice. **b** Row-scaled gene expression of marker genes (35-37) involved in phagocytosis, per subcluster in 14 dpi and naïve datasets.

Figure S6

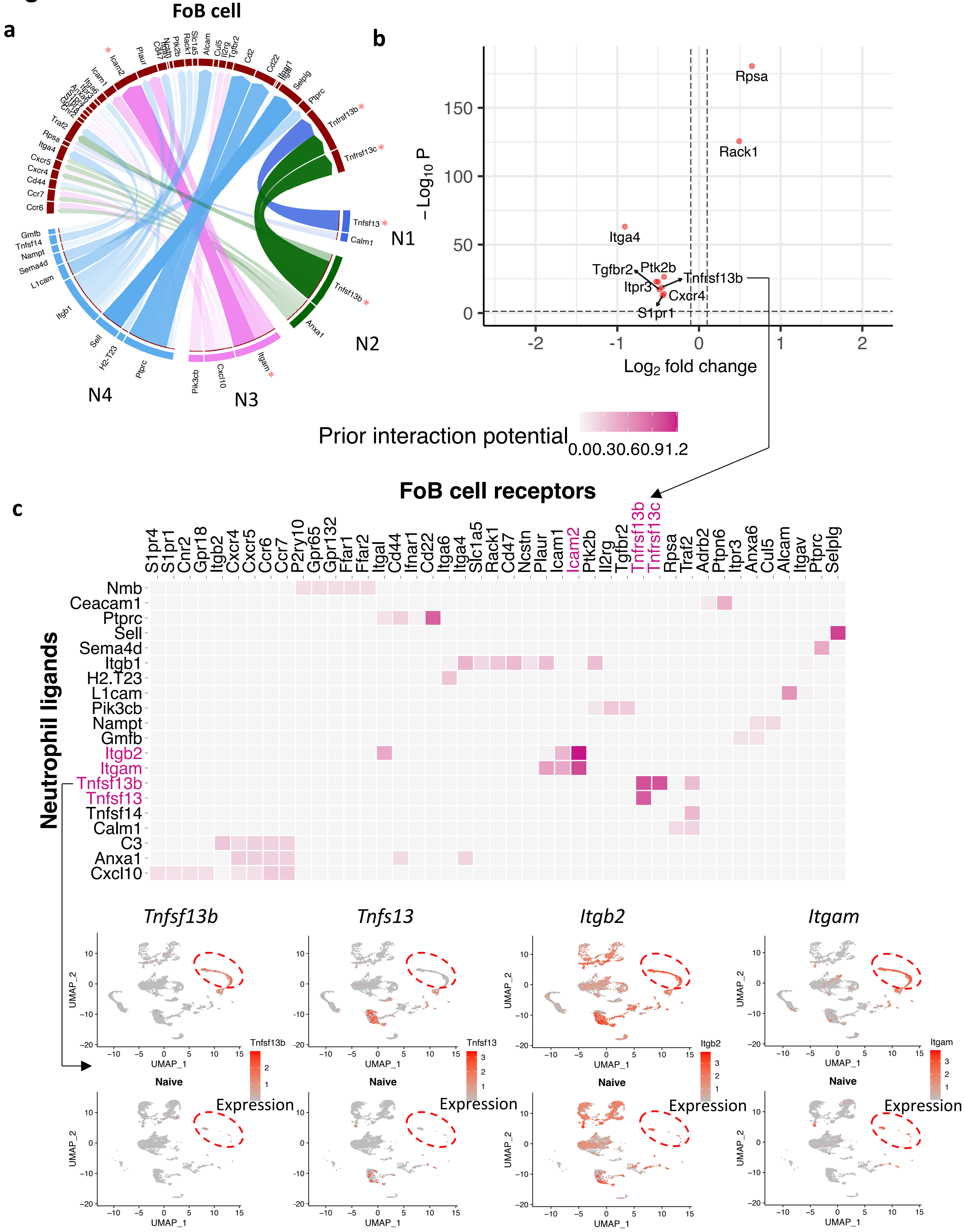

a
FoB cell
N1
N2
N3
N4
b
Rpsa
Rack1
Itga4
Tgfbr2
Ptk2b
Itpr3
Tnfrsf13b
Cxcr4
S1pr1
Log2 fold change
Prior interaction potential
c
FoB cell receptors
Neutrophil ligands
Nmb
Ceacam1
Ptprc
Sell
Sema4d
Itgb1
H2.T23
L1cam
Pik3cb
Nampt
Gmfb
Itgb2
Itgam
Tnfsf13b
Tnfsf13
Tnfsf14
Calm1
C3
Anxa1
Cxcl10
Tnfsf13b
Tnfs13
Itgb2
Itgam
UMAP_1
UMAP_2
Naive
Expression

Supplementary Fig. s6. Crosstalk analysis between neutrophils and FoBs. a Circos plot of ligand-receptor interactions between FoB cells (dark red) and neutrophils N1 (royal blue), N2 (dark green), N3 (violet) and N4 (steel blue). Genes representing a major pathway BAFF (*Tnfsf13b*), APRIL (*Tnfsf13*), TACI (*Tnfrsf13b*), BAFF-R (*Tnfrsf13c*) have been marked (*). **b** Volcano plot of differential gene expression analysis between FoBs of 14 dpi and naïve mice created using the *EnhancedVolcano* package in R. Each dot represents a gene, with the x-axis showing the log2 fold change between conditions, and the y-axis displaying the -log10 *p*-value from the differential expression analysis. Genes with statistically significant differential expression (*p*-value <0.05) and a log2 fold change greater than 1 or less than -1 are colored in red. Genes that did not meet these criteria are shown in green. **c** Heatmap of the prior interaction potential specific for neutrophil ligands and FoB cell receptors. UMAP feature plot representing the expression level of *Tnfsf13b* (BAFF), *Tnfsf13* (APRIL), *Itgb2* (CD18) and *Itgam* (CD11b) in neutrophils of the naïve and 14 dpi mouse spleen samples by scRNA-seq.

Figure S7

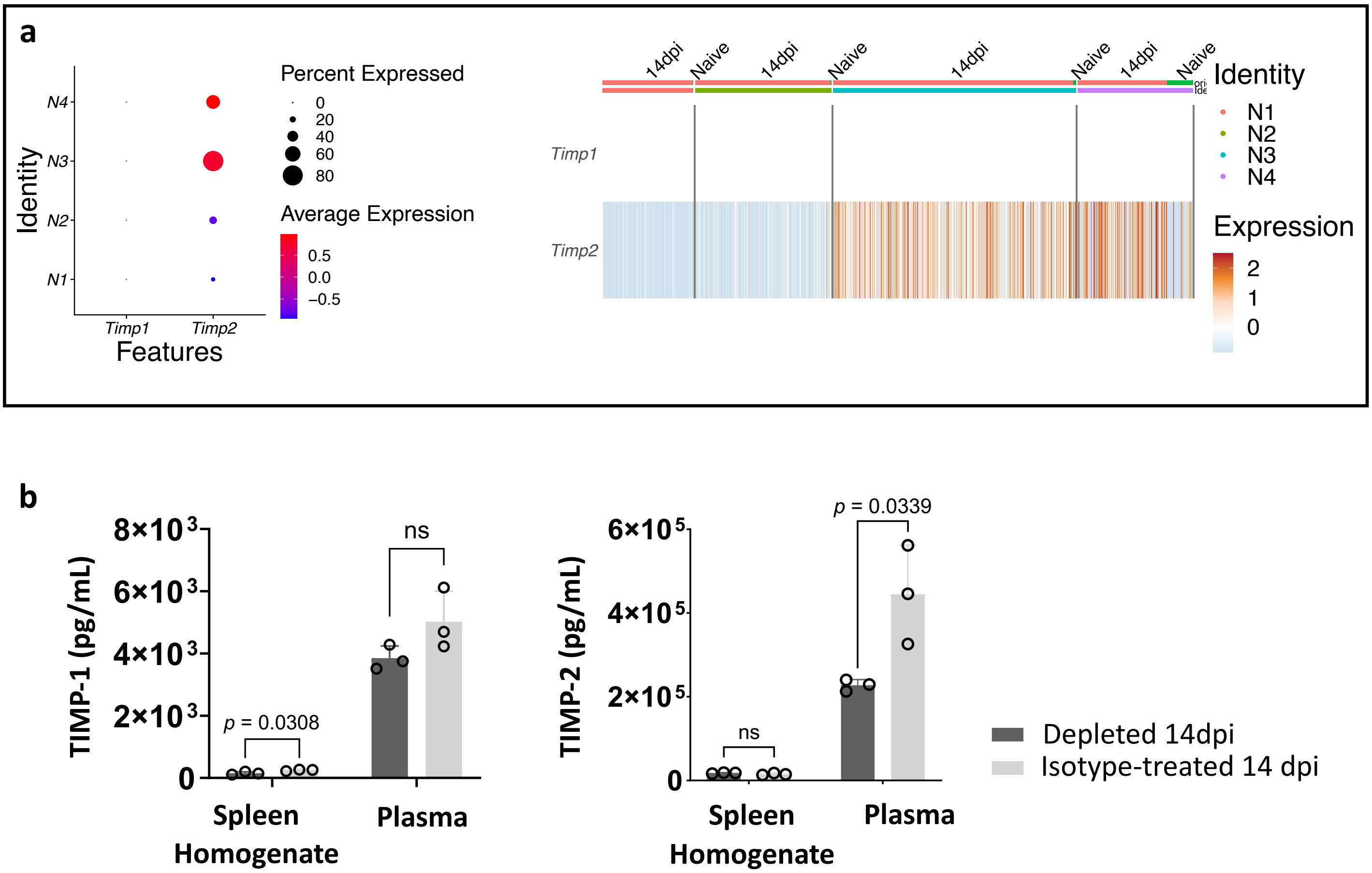

Supplementary Fig. s7. TIMP expression and production during *T. brucei* infection. a Dot plot representing average expression of *Timp1* and *Timp2* across each subcluster. Dot size shows the percentage of cells in each subcluster with more than one read of the corresponding gene. Row-scaled gene expression of *Timp1* and *Timp2* genes in four neutrophil subclusters (N1-N4) of 14 dpi and naïve mice. **b** Concentrations TIMP1 and TIMP2 in spleen extracellular homogenates and plasma were determined for neutrophil-depleted/infected (dark gray) and isotype treated/infected (light gray) mice, using ELISA. Data represent means ± S.D. from one of three representative experiments, with 3 mice per experimental group. Unpaired two-tailed Student's *t*-tests were employed for comparison between experimental groups, with ns indicating non-significant differences ($p \geq 0.05$), and $p < 0.05$ considered as statistically significant. Source data are provided as a Source Data file.

Figure S8

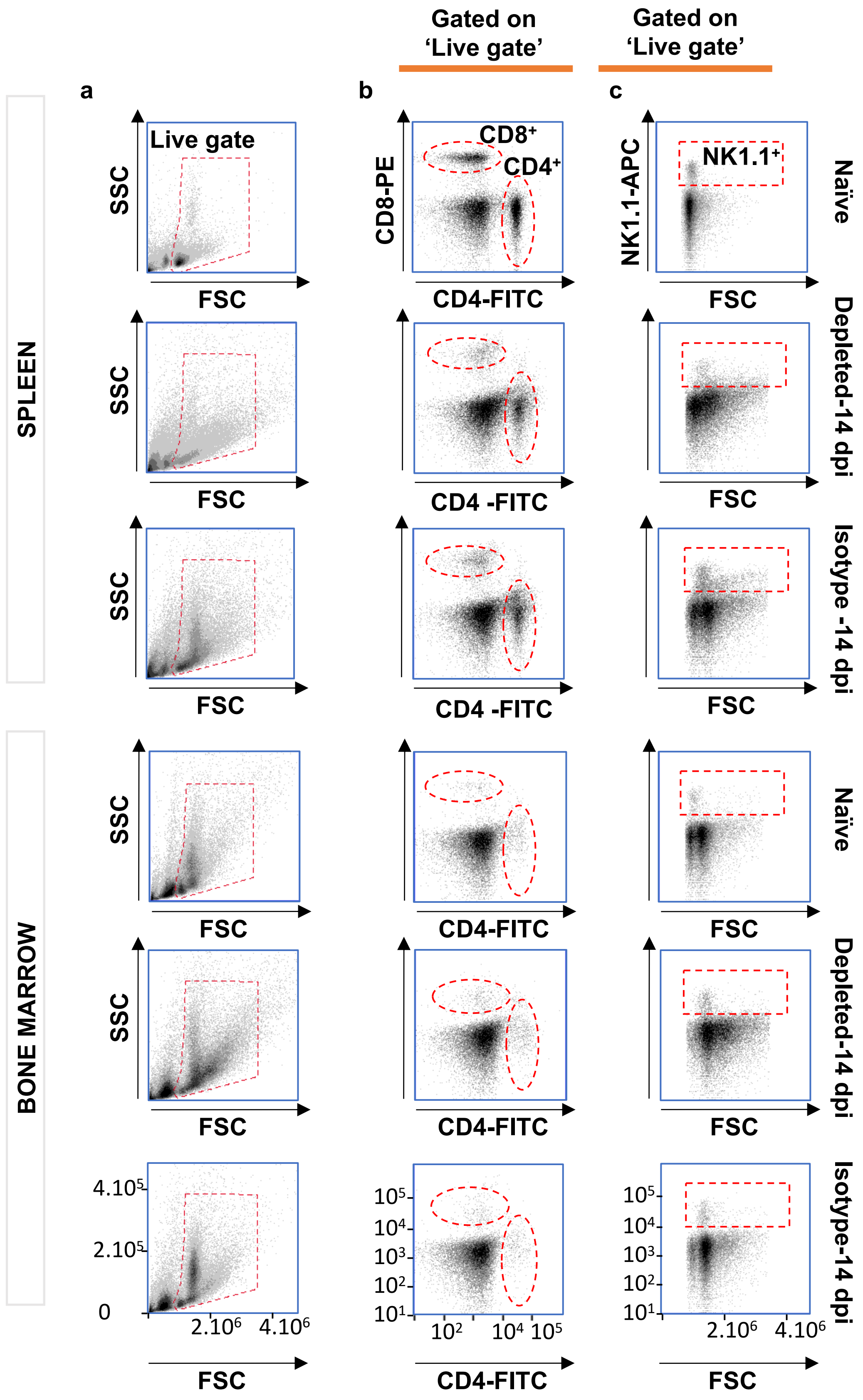

Gated on 'Live gate'
Gated on 'Live gate'
a
b
c
SPLEEN
BONE MARROW
Live gate
SSC
FSC
CD8-PE
CD4-FITC
CD8+
CD4+
NK1.1-APC
NK1.1+
Naïve
Depleted-14 dpi
Isotype -14 dpi
Isotype-14 dpi
4.10^5
2.10^5
0
2.10^6
4.10^6
10^1
10^2
10^3
10^4
10^5

Supplementary Fig. s8. Representative flow cytometry gating strategy for spleen and bone marrow cells including $CD4^{+}$T cells, $CD8^{+}$ T cells and $NK1.1^{+}$ cells. From top to bottom samples represent spleen and bone marrow samples from naïve, 14dpi neutrophil depleted and 14 dpi isotype antibody treated mice. From the left to the right samples represent; **a** FSC/SSC to eliminate debris and red blood cells from the 'Live gate'. **b** CD4/CD8 plot gated on 'Live gate' showing both $CD4^{+}$ T Cells and $CD8^{+}$ T Cells. **c** FSC/NK1.1 plot gated on 'Live gate' showing $NK1.1^{+}$ NKT/NK Cells. Scaling of axes for all graphs in each column is indicated in the lower panel.

Figure S9

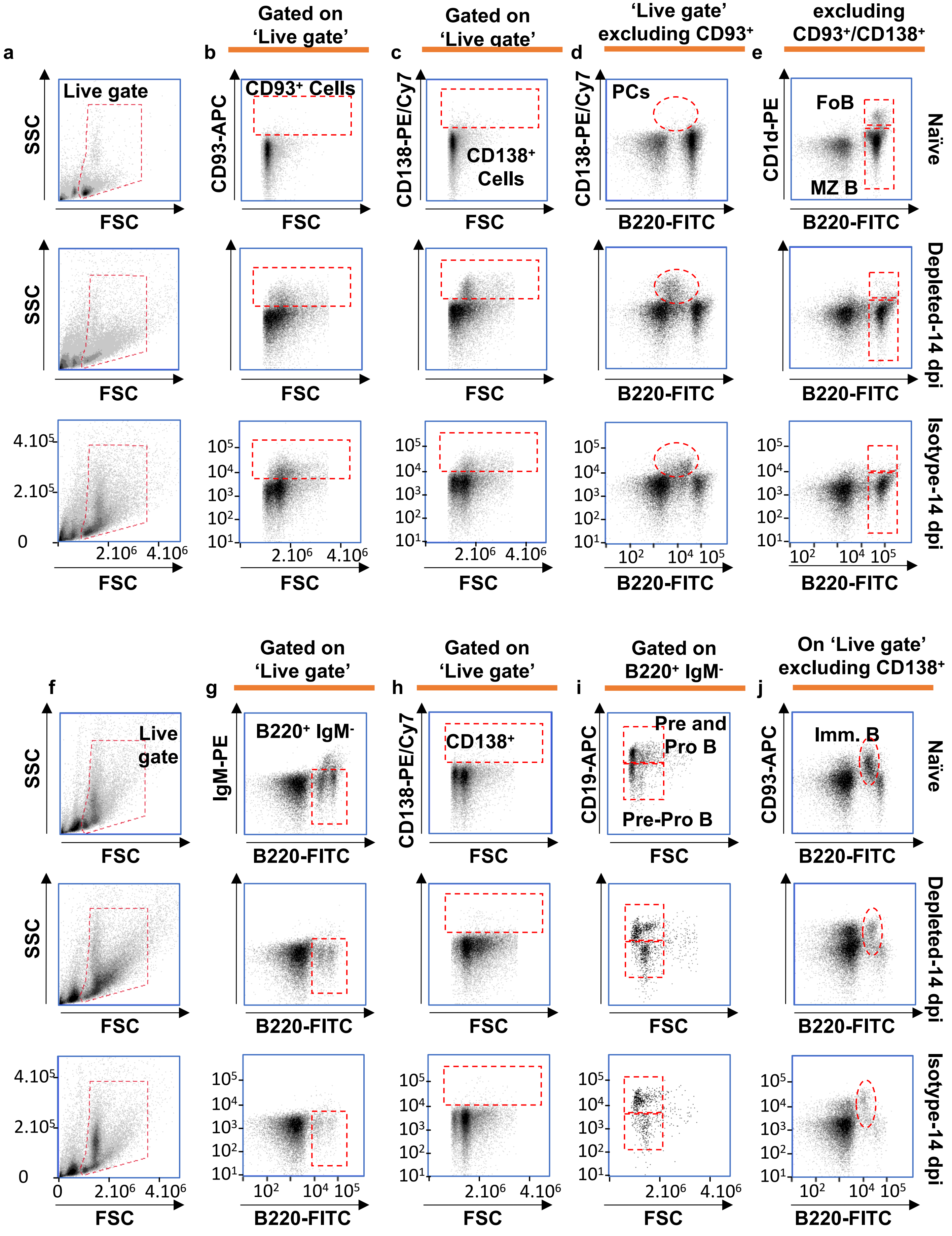
a
b
c
d
e
Gated on 'Live gate'
Gated on 'Live gate'
Gated on 'Live gate' excluding CD93⁺
On 'Live gate' excluding CD93⁺/CD138⁺
Live gate
CD93⁺ Cells
CD138⁺ Cells
PCs
FoB
MZ B
SSC
FSC
CD93-APC
CD138-PE/Cy7
CD1d-PE
B220-FITC
Naive
Depleted-14 dpi
Isotype-14 dpi
f
g
h
i
j
Gated on 'Live gate'
Gated on 'Live gate'
Gated on B220⁺ IgM⁻
On 'Live gate' excluding CD138⁺
Live gate
B220⁺ IgM⁻
CD138⁺
Pre and Pro B
Pre-Pro B
Imm. B
IgM-PE
CD19-APC
CD93-APC

Supplementary Fig. s9. Representative flow cytometry gating strategy for splenic B cells including Marginal zone B cells (MZBs), Follicular B cells (FoBs) and Plasma cells (PCs) and bone marrow B cells including Pre-Pro B cells, Pre and Pro B cells and Immature B cells. From top to bottom samples represent samples from naïve, 14dpi neutrophil depleted and 14 dpi isotype antibody treated mice. From the left to the right samples represent **a** FSC/SSC to eliminate debris and red blood cells from the 'Live gate". **b** FSC/CD93 to define immature 'Live' $CD93^+$ cells. **c** FSC/CD138 to define the 'Live' $CD138^+$ cell population. **d** B220/CD138 plot gated on the 'Live gate' excluding '$CD93^+$ cells' to define $CD138^+$ PCs. **e** B220/CD1d plot with two gates showing both $B220^+CD1d^{High}$ MZBs and $B220^+CD1d^{Low}$ FoBs. Cells in this plot were gated in the 'Live gate' excluding $CD93^+$ and $CD138^+$ cells. Scaling of axes for all graphs in each column is indicated in the lower panel. **f** FSC/SSC to eliminate debris and red blood cells from the 'Live gate'. **g** B220/IgM plot to defining 'Live' $B220^+IgM^-$ cells. **h** FSC/CD138 to define the 'Live' $CD138^+$ cell population. **i** FSC/CD19 plot gated on 'Live' $B220^+IgM^-$ showing both $B220^+IgM^-CD19^-$ Pre-Pro B cells and $B220^+IgM^-CD19^+$ Pre and Pro B cells. **j** B220/CD93 plot gated on the 'Live gate' excluding $CD138^+$ cells, defining Immature B Cells. Scaling of axes for all graphs in each column is indicated in the lower panel.
