# Supplementary material for: Neutrophil metalloproteinase driven spleen damage hampers infection control of trypanosomiasis
Source: Nat Commun. 2023 Sep 5;14:5418. doi: 10.1038/s41467-023-41089-w (PMC10480172; doi:10.1038/s41467-023-41089-w)
Supplement: Supplementary file 3 — Reporting Summary [file 41467_2023_41089_MOESM3_ESM.pdf]

nature portfolio

Corresponding author(s): Magdalena Radwanska

Last updated by author(s): 2023-08-12

Reporting Summary

Nature Portfolio wishes to improve the reproducibility of the work that we publish. This form provides structure for consistency and transparency in reporting. For further information on Nature Portfolio policies, see our Editorial Policies and the Editorial Policy Checklist.

Statistics

For all statistical analyses, confirm that the following items are present in the figure legend, table legend, main text, or Methods section.

n/a	Confirmed	
☐	☑	The exact sample size (*n*) for each experimental group/condition, given as a discrete number and unit of measurement
☐	☑	A statement on whether measurements were taken from distinct samples or whether the same sample was measured repeatedly
☐	☑	The statistical test(s) used AND whether they are one- or two-sided<br>*Only common tests should be described solely by name; describe more complex techniques in the Methods section.*
☑	☐	A description of all covariates tested
☐	☑	A description of any assumptions or corrections, such as tests of normality and adjustment for multiple comparisons
☐	☑	A full description of the statistical parameters including central tendency (e.g. means) or other basic estimates (e.g. regression coefficient) AND variation (e.g. standard deviation) or associated estimates of uncertainty (e.g. confidence intervals)
☐	☑	For null hypothesis testing, the test statistic (e.g. *F*, *t*, *r*) with confidence intervals, effect sizes, degrees of freedom and *P* value noted<br>*Give P values as exact values whenever suitable.*
☑	☐	For Bayesian analysis, information on the choice of priors and Markov chain Monte Carlo settings
☑	☐	For hierarchical and complex designs, identification of the appropriate level for tests and full reporting of outcomes
☑	☐	Estimates of effect sizes (e.g. Cohen's *d*, Pearson's *r*), indicating how they were calculated

Our web collection on statistics for biologists contains articles on many of the points above.

Software and code

Policy information about availability of computer code

Data collection

Data collection for scRNAseq was performed by Illumina Novaseq 6000 sequencer, a mouse reference mouse (mmu10) and Cell Ranger software (v5.0.0 for Spleen samples or v7.0.1 for BM samples.

Data analysis

Analysis performed in R version 4.0.3, Seurat v4.0.5 (Spleen) & v4.3.0 (BM), Monocle v3, SingleR v1.4.1 (Spleen) & v2.0.0 (BM), Harmony version 0.1.1, DoMultiBarHeatmap version 0.1.0, Nichnetr version 1.0.0. Flow Cytometry data was analyzed using BD Accuri TM C6 software, version 1.0.
The codes generated during this study are available at Github repository and can be archived at Zenodo.org:
- For spleen: http://doi.org/10.5281/zenodo.8232243
- For BM: http://doi.org/10.5281/zenodo.8232730

For manuscripts utilizing custom algorithms or software that are central to the research but not yet described in published literature, software must be made available to editors and reviewers. We strongly encourage code deposition in a community repository (e.g. GitHub). See the Nature Portfolio guidelines for submitting code & software for further information.

Data

Policy information about availability of data

All manuscripts must include a data availability statement. This statement should provide the following information, where applicable:

- Accession codes, unique identifiers, or web links for publicly available datasets
- A description of any restrictions on data availability
- For clinical datasets or third party data, please ensure that the statement adheres to our policy

All raw single-cell RNA sequencing and processed data can be accessed from NCBI Gene Expression Omnibus database
- For Spleen: accession code GSE222784 (including GSM6932272 and GSM6932273 for Naïve and 14 dpi dataset, respectively).
- For BM: GSE234000

Human research participants

Policy information about studies involving human research participants and Sex and Gender in Research.

Reporting on sex and gender	No human samples or subjects were used in this study
Population characteristics	No human samples or subjects were used in this study
Recruitment	No human samples or subjects were used in this study
Ethics oversight	No human samples or subjects were used in this study

Note that full information on the approval of the study protocol must also be provided in the manuscript.

Field-specific reporting

Please select the one below that is the best fit for your research. If you are not sure, read the appropriate sections before making your selection.

☑ Life sciences ☐ Behavioural & social sciences ☐ Ecological, evolutionary & environmental sciences

For a reference copy of the document with all sections, see nature.com/documents/nr-reporting-summary-flat.pdf

Life sciences study design

All studies must disclose on these points even when the disclosure is negative.

Sample size	The sample size of every individual group within every single experiment is indicated in the individual figure legends. ScRNA sequencing were performed in 1 sample for each group due to the limitation of the technology and budget. For flow cytometry, ELISA and histology experiment, a sample size of at least 3 mice per experimental condition were conducted. Sample sizes were determined based on the feasibility of obtaining sufficient biological replicates to ensure robustness and statistical significance of our findings. While no formal sample size calculation was conducted due to the exploratory nature of this study and the complexity of single-cell analyses, we adopted a sample size that aligns with common practices in similar. Specifically, the sample sizes were chosen to ensure a representative capture of biological variability and the ability to identify significant differences in gene expression patterns across the investigated conditions.
Data exclusions	No data were excluded from the analysis
Replication<br>Randomization	Randomization of scRNAseq was not appropriate as both naive and infected mice were processed in parallel. The allocation of samples into experimental groups was not randomized due to the nature of the study, which involved time course analysis of infection progression. Instead, efforts were made to control potential confounding factors by ensuring uniformity in the age, sex, and genetic background of the mice used in each group. Additionally, the allocation of samples was based on the availability of animals and the need to achieve a balanced representation of both experimental conditions. Our focus has been on identifying consistent trends and significant patterns across experimental replicates, which allows us to confidently interpret the observed outcomes.
Blinding	Blinding was not performed in this study due to the nature of the experimental design. The analyses involved automated data processing pipelines and computational algorithms, minimizing the potential for subjective bias. Additionally, the primary outcomes measured were quantitative and objective, reducing the likelihood of bias in result interpretation. While blinding was not implemented, rigorous quality control measures and standardized procedures were employed to ensure the reliability and reproducibility of the results.

Behavioural & social sciences study design

All studies must disclose on these points even when the disclosure is negative.

Study description	*Briefly describe the study type including whether data are quantitative, qualitative, or mixed-methods (e.g. qualitative cross-sectional, quantitative experimental, mixed-methods case study).*
Research sample	*State the research sample (e.g. Harvard university undergraduates, villagers in rural India) and provide relevant demographic information (e.g. age, sex) and indicate whether the sample is representative. Provide a rationale for the study sample chosen. For studies involving existing datasets, please describe the dataset and source.*
Sampling strategy	*Describe the sampling procedure (e.g. random, snowball, stratified, convenience). Describe the statistical methods that were used to predetermine sample size OR if no sample-size calculation was performed, describe how sample sizes were chosen and provide a rationale for why these sample sizes are sufficient. For qualitative data, please indicate whether data saturation was considered, and what criteria were used to decide that no further sampling was needed.*

Data collection	*Provide details about the data collection procedure, including the instruments or devices used to record the data (e.g. pen and paper, computer, eye tracker, video or audio equipment) whether anyone was present besides the participant(s) and the researcher, and whether the researcher was blind to experimental condition and/or the study hypothesis during data collection.*
Timing	*Indicate the start and stop dates of data collection. If there is a gap between collection periods, state the dates for each sample cohort.*
Data exclusions	*If no data were excluded from the analyses, state so OR if data were excluded, provide the exact number of exclusions and the rationale behind them, indicating whether exclusion criteria were pre-established.*
Non-participation	*State how many participants dropped out/declined participation and the reason(s) given OR provide response rate OR state that no participants dropped out/declined participation.*
Randomization	*If participants were not allocated into experimental groups, state so OR describe how participants were allocated to groups, and if allocation was not random, describe how covariates were controlled.*

Ecological, evolutionary & environmental sciences study design

All studies must disclose on these points even when the disclosure is negative.

Study description	*Briefly describe the study. For quantitative data include treatment factors and interactions, design structure (e.g. factorial, nested, hierarchical), nature and number of experimental units and replicates.*
Research sample	*Describe the research sample (e.g. a group of tagged Passer domesticus, all Stenocereus thurberi within Organ Pipe Cactus National Monument), and provide a rationale for the sample choice. When relevant, describe the organism taxa, source, sex, age range and any manipulations. State what population the sample is meant to represent when applicable. For studies involving existing datasets, describe the data and its source.*
Sampling strategy	*Note the sampling procedure. Describe the statistical methods that were used to predetermine sample size OR if no sample-size calculation was performed, describe how sample sizes were chosen and provide a rationale for why these sample sizes are sufficient.*
Data collection	*Describe the data collection procedure, including who recorded the data and how.*
Timing and spatial scale	*Indicate the start and stop dates of data collection, noting the frequency and periodicity of sampling and providing a rationale for these choices. If there is a gap between collection periods, state the dates for each sample cohort. Specify the spatial scale from which the data are taken*
Data exclusions	*If no data were excluded from the analyses, state so OR if data were excluded, describe the exclusions and the rationale behind them, indicating whether exclusion criteria were pre-established.*
Reproducibility	*Describe the measures taken to verify the reproducibility of experimental findings. For each experiment, note whether any attempts to repeat the experiment failed OR state that all attempts to repeat the experiment were successful.*
Randomization	*Describe how samples/organisms/participants were allocated into groups. If allocation was not random, describe how covariates were controlled. If this is not relevant to your study, explain why.*
Blinding	*Describe the extent of blinding used during data acquisition and analysis. If blinding was not possible, describe why OR explain why blinding was not relevant to your study.*

Did the study involve field work? ☐ Yes ☐ No

Field work, collection and transport

Field conditions	*Describe the study conditions for field work, providing relevant parameters (e.g. temperature, rainfall).*
Location	*State the location of the sampling or experiment, providing relevant parameters (e.g. latitude and longitude, elevation, water depth).*
Access & import/export	*Describe the efforts you have made to access habitats and to collect and import/export your samples in a responsible manner and in compliance with local, national and international laws, noting any permits that were obtained (give the name of the issuing authority, the date of issue, and any identifying information).*
Disturbance	*Describe any disturbance caused by the study and how it was minimized.*

Reporting for specific materials, systems and methods

We require information from authors about some types of materials, experimental systems and methods used in many studies. Here, indicate whether each material, system or method listed is relevant to your study. If you are not sure if a list item applies to your research, read the appropriate section before selecting a response.

Materials & experimental systems

n/a	Involved in the study
☐	☑ Antibodies
☑	☐ Eukaryotic cell lines
☑	☐ Palaeontology and archaeology
☐	☑ Animals and other organisms
☑	☐ Clinical data
☑	☐ Dual use research of concern

Methods

n/a	Involved in the study
☑	☐ ChIP-seq
☐	☑ Flow cytometry
☑	☐ MRI-based neuroimaging

Antibodies

Antibodies used

The following anti-mouse antibodies were used:

- For Flow Cytometry: Alexa Fluor ® 488 anti-mouse Ly-6G (BioLegend, 127626, clone 1A8), PE anti-mouse Ly-6G (BioLegend, 127607, clone 1A8), PE/Cyanine7 anti-mouse Ly-6C (BioLegend, 128017, clone HK1.4), FITC anti-mouse/human CD11b (BioLegend, 101206, clone M1/70), APC anti-mouse/human CD11b (BioLegend, 101212, clone M1/70), PE anti-mouse CD182 (CXCR2) (BioLegend, 149303, clone SA044G4), PE anti-mouse CD184 (CXCR4) (BioLegend, 146505, clone L276F12), Alexa Fluor® 647 rat anti-mouse CD177 (BD Biosciences, 566599, clone Y127), FITC anti-mouse CD4 (BioLegend, 100406, clone GK1.5), PE anti-mouse CD8a (BioLegend, 100708, clone 53-6.7), APC anti-mouse NK-1.1 (BioLegend, 108710, clone PK136), FITC anti-mouse/human CD45R/B220 (BioLegend, 103206, clone RA3-6B2), PE anti-mouse/human CD45R/B220 (BioLegend, 103208, clone RA3-6B2), PE anti-mouse CD1d (CD1.1, Ly-38) (BioLegend, 123510, clone 1B1), PE anti-mouse IgM (BioLegend, 406507, clone RMM-1), PE/Cyanine7 anti-mouse CD138 (Syndecan-1) (BioLegend, 142514, clone 281-2), PE/Cyanine7 anti-mouse CD93 (AA4.1, early B lineage) (BioLegend, 136506, clone AA4.1), APC anti-mouse CD93 (AA4.1, early B lineage) (BioLegend, 136510, clone AA4.1), APC anti-mouse CD23 (BioLegend, 101619, clone B3B4), APC anti-mouse CD19 (BioLegend, 152409, clone 1D3/CD19), FITC anti-mBCMA (R&D System, TAB593F, clone 161616).
- For Histology study: Alexa Fluor ® 488 anti-mouse Ly-6G (BioLegend, 127626, clone 1A8), Anti-Myeloperoxidase antibody (abcam, ab90810, clone 2D4), Neutrophil Elastase Polyclonal Antibody, Phycoerythrin Conjugated (Bioss, BS-6982R-PE), Recombinant Anti-Histone H2B antibody (abcam, ab52599, clone EP957Y), Rabbit anti Mouse Collagen I (Bio-Rad, 2150-1410), Elastin Polyclonal Antibody (Bioss, BS-1756R), Cyanine3 Streptavidin (BioLegend, 405215), Goat Anti-Rabbit IgG H&L (Alexa Fluor® 488) (abcam, ab150077).

Validation

All antibodies used in this study were validated for species specificity and application suitability. Validation information, including specificity and recommended applications, was obtained from the manufacturer's websites and technical documentation. Concentration were decided according to recommended by manufacturer and tested in pilot titration experiments.

Eukaryotic cell lines

Policy information about cell lines and Sex and Gender in Research

Cell line source(s)

State the source of each cell line used and the sex of all primary cell lines and cells derived from human participants or vertebrate models.

Authentication

Describe the authentication procedures for each cell line used OR declare that none of the cell lines used were authenticated.

Mycoplasma contamination

Confirm that all cell lines tested negative for mycoplasma contamination OR describe the results of the testing for mycoplasma contamination OR declare that the cell lines were not tested for mycoplasma contamination.

Commonly misidentified lines (See ICLAC register)

Name any commonly misidentified cell lines used in the study and provide a rationale for their use.

Palaeontology and Archaeology

Specimen provenance

Provide provenance information for specimens and describe permits that were obtained for the work (including the name of the issuing authority, the date of issue, and any identifying information). Permits should encompass collection and, where applicable, export.

Specimen deposition

Indicate where the specimens have been deposited to permit free access by other researchers.

Dating methods

If new dates are provided, describe how they were obtained (e.g. collection, storage, sample pretreatment and measurement), where they were obtained (i.e. lab name), the calibration program and the protocol for quality assurance OR state that no new dates are provided.

☐ Tick this box to confirm that the raw and calibrated dates are available in the paper or in Supplementary Information.

Ethics oversight

Identify the organization(s) that approved or provided guidance on the study protocol, OR state that no ethical approval or guidance was required and explain why not.

Note that full information on the approval of the study protocol must also be provided in the manuscript.

Animals and other research organisms

Policy information about studies involving animals; ARRIVE guidelines recommended for reporting animal research, and Sex and Gender in Research

Laboratory animals	Adult female (6-8 week old) female C57BL/6 (WT) mice were purchased from Koatech (South Korea). Animals were housed in IVCs with appropriate cage enrichment and fed ad libitum. A 12-hour light/dark cycle was maintained, with ambient temperature at 20-24°C and humidity levels between 40-60%.
Wild animals	No wild animals were used in this study
Reporting on sex	Only female mice were used in this study. Female mice are often used in research because they are less aggressive than males, reducing the likelihood of injuries that could affect experimental results. Their social structure (they can be housed in groups without fighting) also makes it easier to manage them in a laboratory setting. Additionally, the estrous cycle in female mice is much shorter and more regular than the menstrual cycle , offering more consistency in experimental conditions
Field-collected samples	No field samples were used in this study
Ethics oversight	All experimental animal procedures were approved by the GUGC Institutional Animal Care and Use Committee (IACUC), file numbers 2019-011, 2019.019.A, 2019-025, 2020-009, 2020-018, 2021-005, 2022-001, 2022-007, 2022-012, 2023-005, 2023-008, 2023-009 and 2023-012 All experimental animal procedures were conducted according to EU directive 2010/63/EU.

Note that full information on the approval of the study protocol must also be provided in the manuscript.

Clinical data

Policy information about clinical studies

All manuscripts should comply with the ICMJE guidelines for publication of clinical research and a completed CONSORT checklist must be included with all submissions.

Clinical trial registration	*Provide the trial registration number from ClinicalTrials.gov or an equivalent agency.*
Study protocol	*Note where the full trial protocol can be accessed OR if not available, explain why.*
Data collection	*Describe the settings and locales of data collection, noting the time periods of recruitment and data collection.*
Outcomes	*Describe how you pre-defined primary and secondary outcome measures and how you assessed these measures.*

Dual use research of concern

Policy information about dual use research of concern

Hazards

Could the accidental, deliberate or reckless misuse of agents or technologies generated in the work, or the application of information presented in the manuscript, pose a threat to:

No	Yes	
☐	☐	Public health
☐	☐	National security
☐	☐	Crops and/or livestock
☐	☐	Ecosystems
☐	☐	Any other significant area

Experiments of concern

Does the work involve any of these experiments of concern:

No	Yes	
☐	☐	Demonstrate how to render a vaccine ineffective
☐	☐	Confer resistance to therapeutically useful antibiotics or antiviral agents
☐	☐	Enhance the virulence of a pathogen or render a nonpathogen virulent
☐	☐	Increase transmissibility of a pathogen
☐	☐	Alter the host range of a pathogen
☐	☐	Enable evasion of diagnostic/detection modalities
☐	☐	Enable the weaponization of a biological agent or toxin
☐	☐	Any other potentially harmful combination of experiments and agents

ChIP-seq

Data deposition

☐ Confirm that both raw and final processed data have been deposited in a public database such as GEO.

☐ Confirm that you have deposited or provided access to graph files (e.g. BED files) for the called peaks.

Data access links<br>*May remain private before publication.*	*For "Initial submission" or "Revised version" documents, provide reviewer access links. For your "Final submission" document, provide a link to the deposited data.*
Files in database submission	*Provide a list of all files available in the database submission.*
Genome browser session<br>(e.g. UCSC)	*Provide a link to an anonymized genome browser session for "Initial submission" and "Revised version" documents only, to enable peer review. Write "no longer applicable" for "Final submission" documents.*

Methodology

Replicates	*Describe the experimental replicates, specifying number, type and replicate agreement.*
Sequencing depth	*Describe the sequencing depth for each experiment, providing the total number of reads, uniquely mapped reads, length of reads and whether they were paired- or single-end.*
Antibodies	*Describe the antibodies used for the ChIP-seq experiments; as applicable, provide supplier name, catalog number, clone name, and lot number.*
Peak calling parameters	*Specify the command line program and parameters used for read mapping and peak calling, including the ChIP, control and index files used.*
Data quality	*Describe the methods used to ensure data quality in full detail, including how many peaks are at FDR 5% and above 5-fold enrichment.*
Software	*Describe the software used to collect and analyze the ChIP-seq data. For custom code that has been deposited into a community repository, provide accession details.*

Flow Cytometry

Plots

Confirm that:

☑ The axis labels state the marker and fluorochrome used (e.g. CD4-FITC).

☑ The axis scales are clearly visible. Include numbers along axes only for bottom left plot of group (a 'group' is an analysis of identical markers).

☑ All plots are contour plots with outliers or pseudocolor plots.

☑ A numerical value for number of cells or percentage (with statistics) is provided.

Methodology

Sample preparation

Mouse spleens were isolated after CO2 euthanasia of the animals. Single-cell suspensions were prepared by homogenizing spleens in 4 mL of Dulbecco's Modified Eagle Medium (DMEM) supplemented with 10% Fetal Bovine Serum (FBS) and 1% penicillin/streptomycin using gentleMACS™ Dissociator. After passing the homogenate through a 70 µm cell strainer, cells were centrifuged at 314 x g at 4oC for 7 minutes. Cell pellets were resuspended in RBC lysis buffer at 4oC and incubated for 5 minutes. After washing (314 x g at 4oC for 7 minutes), cells were kept in FACSFlow Sheath Fluid containing 0.05% FBS on ice. Total remaining live cells were counted by Trypan Blue. Supernatant fraction collected after centrifugation of tissue homogenate were centrifuged once more at 10,000 x g for 10 min at 4oC. Final supernatant was recuperated and frozen at -20oC for further analysis.
Bone marrow femur cells were collected from CO2 euthanized mice by isolating and flushing femurs, using a 26-gauge needle and 1 mL ice-cold DMEM supplemented with 1% FBS and 1% penicillin/streptomycin. Cell suspensions were centrifuged at 314 x g at 4oC for 7 mins. Cell pellets were re-suspended in 3 mL FACSFlow Sheath Fluid containing 0.05% FBS and total remaining live cells were counted using Trypan Blue.
Blood was collected from CO2 euthanized mice by cardiac puncture, with a 1mL syringe filled with 0.1 mL heparin (500 IU/mL). Samples were subsequently mixed with 20 mL RBC lysis buffer at 4oC for 15 minutes. After two wash steps with 20 mL ice-cold DMEM (centrifugation at 314g at 4oC for 7 minutes), blood cells were re-suspended in 1 mL of FACSFlow Sheath Fluid containing 0.05% FBS on ice. Total remaining live cells were counted by Trypan Blue). For plasma preparation, heparinized blood samples were centrifuged at 2,000 x g at 4oC for 10 min. Next, plasma was aliquoted into 3 Eppendorf tubes per mouse and stored at -20oC for further analysis.
Cell staining was performed in FACSFlow Sheath Fluid containing 0.05% FBS using 105 cells per sample. Nonspecific binding was blocked using 1/1000 diluted CD16/CD32 Fcγ III/II (Biolegend, CA, USA) for 20 minutes in the dark at 4oC. Subsequently, cells were incubated with predetermined optimal concentrations (Supplementary Table s1) of fluorochrome-conjugated antibodies and/or isotype controls for 30 minutes in the dark at 4oC.

Instrument	BD Accuri™ C6 Plus flow cytometer (BD Biosciences, CA, USA)
Software	BD AccuriTM C6 software, version 1.0 (BD Biosciences, CA, USA)

Cell population abundance

Up to 100,000 events were collected

Gating strategy

Initial gates were set based on forward scatter (FSC) and side scatter (SSC) parameters to encompass the starting cell population. Boundaries between "positive" and "negative" staining cell populations were defined based on fluorescence intensity levels and isotype controls. These strategies were consistently applied across relevant experiments to ensure precise data interpretation. Downstream cell types are defined as following gating strategy: Ly6G^{+}CD177^{+}Ly6C^{+}CD11b^{+} Neutrophils, CD11b^{+}Ly6C^{+} Monocyte, B220^{+}IgM^{-}CD19^{-} Pre-Pro B cells, B220^{+}IgM^{-}CD19^{+} Pro and Pre B cells, B220^{+}CD93^{+} Immature B cells, B220^{+}Cd1d^{High} MZ B cells, B220+Cd1d^{Low} Fo B cells, B220^{Int}CD138^{+} Plasma Cells, CD4^{+} T Cells, CD8^{+} T Cells and NK1.1^{+} NKT/NK Cells.

☑ Tick this box to confirm that a figure exemplifying the gating strategy is provided in the Supplementary Information.

Magnetic resonance imaging

Experimental design

Design type — *Indicate task or resting state; event-related or block design.*

Design specifications — *Specify the number of blocks, trials or experimental units per session and/or subject, and specify the length of each trial or block (if trials are blocked) and interval between trials.*

Behavioral performance measures — *State number and/or type of variables recorded (e.g. correct button press, response time) and what statistics were used to establish that the subjects were performing the task as expected (e.g. mean, range, and/or standard deviation across subjects).*

Acquisition

Imaging type(s) — *Specify: functional, structural, diffusion, perfusion.*

Field strength — *Specify in Tesla*

Sequence & imaging parameters — *Specify the pulse sequence type (gradient echo, spin echo, etc.), imaging type (EPI, spiral, etc.), field of view, matrix size, slice thickness, orientation and TE/TR/flip angle.*

Area of acquisition — *State whether a whole brain scan was used OR define the area of acquisition, describing how the region was determined.*

Diffusion MRI ☐ Used ☐ Not used

Preprocessing

Preprocessing software — *Provide detail on software version and revision number and on specific parameters (model/functions, brain extraction, segmentation, smoothing kernel size, etc.).*

Normalization — *If data were normalized/standardized, describe the approach(es): specify linear or non-linear and define image types used for transformation OR indicate that data were not normalized and explain rationale for lack of normalization.*

Normalization template — *Describe the template used for normalization/transformation, specifying subject space or group standardized space (e.g. original Talairach, MNI305, ICBM152) OR indicate that the data were not normalized.*

Noise and artifact removal — *Describe your procedure(s) for artifact and structured noise removal, specifying motion parameters, tissue signals and physiological signals (heart rate, respiration).*

Volume censoring — *Define your software and/or method and criteria for volume censoring, and state the extent of such censoring.*

Statistical modeling & inference

Model type and settings — *Specify type (mass univariate, multivariate, RSA, predictive, etc.) and describe essential details of the model at the first and second levels (e.g. fixed, random or mixed effects; drift or auto-correlation).*

Effect(s) tested — *Define precise effect in terms of the task or stimulus conditions instead of psychological concepts and indicate whether ANOVA or factorial designs were used.*

Specify type of analysis: ☐ Whole brain ☐ ROI-based ☐ Both

Statistic type for inference (See Eklund et al. 2016) — *Specify voxel-wise or cluster-wise and report all relevant parameters for cluster-wise methods.*

Correction — *Describe the type of correction and how it is obtained for multiple comparisons (e.g. FWE, FDR, permutation or Monte Carlo).*

Models & analysis

n/a	Involved in the study
☐	☐ Functional and/or effective connectivity
☐	☐ Graph analysis
☐	☐ Multivariate modeling or predictive analysis

Functional and/or effective connectivity — *Report the measures of dependence used and the model details (e.g. Pearson correlation, partial correlation, mutual information).*

Graph analysis — *Report the dependent variable and connectivity measure, specifying weighted graph or binarized graph, subject- or group-level, and the global and/or node summaries used (e.g. clustering coefficient, efficiency, etc.).*

Multivariate modeling and predictive analysis — *Specify independent variables, features extraction and dimension reduction, model, training and evaluation metrics.*
